# Efficacy and safety of zuranolone in the treatment of major depressive disorder: a meta-analysis

**DOI:** 10.3389/fnins.2023.1332329

**Published:** 2024-01-16

**Authors:** Shuyu Wang, Wenxing Zhang, Zhang Liu, Tian Zhang, Yi Wang, Weihong Li

**Affiliations:** School of Basic Medical Science, Chengdu University of Traditional Chinese Medicine, Chengdu, China

**Keywords:** zuranolone, SAGE-217, depression, MDD, major depressive disorder

## Abstract

**Objective:**

This study aimed to systematically review zuranolone’s efficacy and safety in treating major depressive disorder (MDD).

**Methods:**

We conducted electronic searches in databases like PubMed, Embase, Cochrane, and Web of Science to identify randomized controlled trials using zuranolone for severe depression from study inception to September 15, 2023. Two independent reviewers screened studies, extracted data, and assessed study quality. Our meta-analysis included four studies with 1,454 patients. The findings showed significant improvements with zuranolone across various measures: Hamilton Depression Rating Scale (HAM-D) scores indicated notable alleviation in depressive symptoms (WMD: −2.03; 95% CI: −2.42 to −1.65); the treatment group’s HAM-D score response rate was significantly higher than the control group’s at day 15 (OR: 1.46, 95% CI: 1.11 to 1.92, *P* = 0.01). The meta-analysis also revealed higher remission rates for the treatment group compared to the control group at day 15 (OR: 1.68, 95% CI: 1.18 to 2.39, *P* = 0.03). Additionally, HAM-A scores on day 15 and MADRS scores on day 15 showed improvement, and HAM-D scores for 30 mg zuranolone on different treatment days exhibited improvement (WMD, −2.55; 95% CI, −3.24 to −1.58; *P* = 0.05). However, analyzing HAM-D scores on day 15 for various zuranolone doses revealed no significant differences. Importantly, zuranolone use was associated with an increased incidence of adverse reactions.

**Results:**

Our meta-analysis included four studies with 1454 patients, showing significant improvements with zuranolone across various measures, including HAM-D scores, HAM-A scores, MADRS scores, and specific HAM-D scores for 30 mg zuranolone on different treatment days. However, no significant differences were found in HAM-D scores on day 15 for various doses of zuranolone.

**Conclusions:**

Our findings suggest that zuranolone is a promising, simple, and convenient treatment for patients with major depressive disorder, offering potential guidance for clinical practice.

## 1 Introduction

Even before the emergence of the coronavirus disease 2019 (COVID-19) pandemic, major depressive disorder (MDD) ranked among the leading global causes of health burden (Patel et al., 2016; GBD 2019 Mental Disorders Collaborators, 2022). The advent of the COVID-19 pandemic has exacerbated many determinants of poor mental health (Pirkis et al., 2021). Studies estimate an additional 53.2 million cases of MDD globally attributable to the COVID-19 pandemic (COVID-19 Mental Disorders Collaborators, 2021). MDD is one of the most common, burdensome, and costly psychiatric conditions affecting adults globally (Cipriani et al., 2018). Characterized by symptoms including a persistent depressed mood and loss of interest or pleasure in activities, among others (Shafiee et al., 2018; Köhler-Forsberg et al., 2019; Gronemann et al., 2020; Riemann et al., 2020), MDD impacts more than 3.8% of the worldwide population, marking it as a significant health issue. Recent research highlights the correlation between depression and compromised neuronal activity in key brain networks such as the central executive network (CEN), default mode network (DMN), and salience network (SN) (Yan et al., 2019). A study comparing acute and long-term outcomes within the Sequential Treatment Protocol for Depression Relief (STAR*D) trial evaluated four successive treatment steps, suggesting a theoretical cumulative response rate of 67% (Rush et al., 2006). Presently, medication stands as the primary approach to managing depression, yet antidepressants have limitations, including slow onset of action, prolonged treatment duration, and high rates of relapse. Moreover, extended use can result in diverse side effects such as sexual dysfunction, weight gain, nausea, and headaches (Moret et al., 2009). Additionally, roughly one-third of individuals with severe depression do not exhibit favorable responses to existing antidepressant medications (Daly et al., 2018). Hence, the development of new antidepressants holds critical importance as a resource for clinicians in addressing severe depression.

Zuranolone, a rapid-acting capsule taken once daily for a 14-day duration, swiftly alleviates depressive symptoms. Its effectiveness begins within 3°days, a notable improvement compared to existing treatments that might take weeks or months to show results. Notably, it stands as the first FDA-approved oral medication for postpartum depression, presenting a significant advancement over brexanolone, which is solely available as an intravenous injection administered over 60 h (equivalent to 2.5°days) (Scott, 2019). Zuranolone’s once-daily capsule form provides a more accessible administration method. Operating as a neuroactive steroid (NAS) and GABA-A receptor-positive allosteric modulator (PAM), it is also indicated for severe depression. Its efficacy in treating postpartum severe depression has been established through research focused on postpartum depression.

Gamma-aminobutyric acid (GABA) plays a critical role in maintaining and restoring excitatory-inhibitory balance in the brain while regulating brain networks (Makar et al., 1975). Approximately one-third of neurons in the central nervous system (CNS) are GABAergic, responsible for regulating the function of GABAA receptors both within and outside synapses, thus restoring the balance between inhibitory and excitatory receptors in the brain (Tang et al., 2021). The GABA system serves as a key inhibitory signaling pathway in the brain and CNS and plays an important role in regulating CNS function (Koh et al., 2023). For individuals dealing with depression, zuranolone may facilitate the rapid rebalancing of misaligned neural networks to enhance overall brain function (Carvalho, 2023).

Zuranolone is recognized as a promising antidepressant agent. In clinical trials, treatment with zuranolone has demonstrated significant improvements in depressive symptoms among adults with MDD compared to a placebo, and it has generally exhibited a well-tolerated and consistent safety profile. Currently, the sample size of clinical trials for zuranolone in the treatment of MDD is limited. Therefore, further analysis of a larger dataset is necessary to comprehensively assess its efficacy and safety. Consequently, this article is based on the most recent prospective clinical trials, aiming to objectively evaluate the efficacy and safety of zuranolone in treating patients with MDD and to provide additional evidence for clinical treatment.

## 2 Methods

This meta-analysis was based entirely on the preferred reporting items for systematic reviews and meta-analysis (PRISMA) (Page et al., 2021), following protocols registered at the International Platform of Registered Systematic Review and Meta-Analysis Protocols (INPLASY 20236110116).

### 2.1 Data sources and searches

In this study, we conducted electronic searches in English databases, primarily sourcing relevant literature from PubMed, Embase, Cochrane, and the Web of Science. The search period spanned from the inception of the databases to September 15, 2023. Two researchers (SW and ZL) independently assessed the titles and abstracts of the studies identified during the search, excluding those that were not pertinent. For the remaining studies, we thoroughly examined both the full texts and Supplementary materials to ascertain whether they contained the necessary information. Any disagreements in the study selection process were resolved by referring to the original article and reaching a consensus with the senior investigator (WHL).

### 2.2 Inclusion and exclusion criteria

Inclusion criteria for MDD were as follows: (1) randomized controlled trials (RCT), (2) Diagnostic and Statistical Manual of Mental Disorders, fifth edition (DSM-5) (First, 2013), and 17-item Hamilton Depression Rating Scale (HAM-D) scores (Zimmerman et al., 2013), (3) intervention: zuranolone was administered to the experimental group while the control group received a placebo (Zuranolone, 2023).

Exclusion criteria were as follows: (1) studies with inconsistent subject-object relationships, (2) studies with duplicated data, (3) unavailability of full text or complete data, and (4) studies focusing on MDD subtypes (such as severe postpartum depression or severe post-stroke depression), (5) non-English articles, and (6) publication in the form of letters, conference reports, editorials, case reports, animal studies, basic studies, or systematic reviews.

### 2.3 Data extraction

Endnote 21 was used for literature importation and screening. Two investigators (SW and WZ) conducted the literature screening and data extraction in accordance with the study’s design, as well as the inclusion and exclusion criteria concerning the study participants. Any discrepancies that arose were resolved through discussion until a consensus was achieved. If needed, a third researcher was consulted (ZL). Data obtained from the RCTs included various parameters, including the first author, publication year, sample size, age, gender distribution, the dosage of zuranolone (50, 30, and 20 mg), treatment duration, and outcomes such as HAM-D, MADRS, and HAM-A scores, among other relevant details.

### 2.4 Risk-of-bias assessment

To assess study quality, we utilized the Cochrane Handbook of Systematic Reviews (Cumpston et al., 2019), employing seven key criteria: random sequence generation, allocation concealment, blinding of participants and personnel, blinding of outcome assessment, incomplete outcome data, selective reporting, and other biases. The risk of bias for each criterion was categorized as low, unclear, or high.

### 2.5 Data synthesis and statistical analysis

In this meta-analysis, we designated the HAM-D score on day 15, response and remission rate on day 15 of HAM-D as the primary outcome. The HAM-A score on day 15, the Montgomery-Asberg Depression Rating Scale (MDARS) score on day 15, and the HAM-D score for 30 mg of zuranolone on days 3, 8, and 15 as secondary endings. Additionally, we conducted subgroup analyses for different doses of zuranolone (50, 20, and 30 mg) in assessing HAM-D scores at day 15.

Heterogeneity was evaluated using the chi-square test (*P* < 0.10) and the I squared index (*I^2^* > 50%). When both *P* < 0.05 and *I*^2^ > 50% were met, it indicated substantial heterogeneity among the studies, leading to the adoption of a random effect model. In the analysis of overall effects, we used weighted mean difference (WMD), odds ratio (OR), and 95% confidence interval (95% CI) as the effect indicators. As the number of included studies was <10, the funnel plot and Egger’s test were used to examine the potential presence of publication bias. All analyses were conducted using Comprehensive Meta-Analysis (version 4) for meta-analysis and R software (dosresmeta package version 2.0.1) for dose-response meta-analysis. A significance level of *P* < 0.05 was considered to be statistically significant.

## 3 Results

### 3.1 Literature search results

**FIGURE 1:**
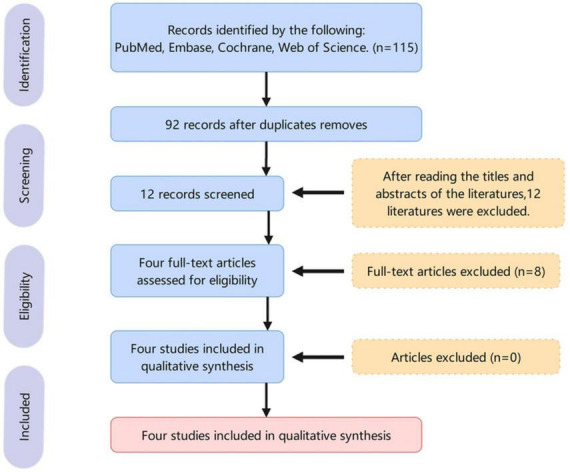
Flowchart of literature selection.

The PRISMA flowchart is presented in Figure 1. The initial search yielded a total of 115 relevant publications. We excluded 92 duplicate articles. Following the screening of the titles and abstracts, we excluded 12 publications. Following a comprehensive evaluation of the full texts in accordance with the inclusion and exclusion criteria, we identified four clinical trials (Gunduz-Bruce et al., 2019; Clayton et al., 2023b; Clayton et al., 2023a; Kato et al., 2023), comprising a total of 1,454 patients with MDD, for inclusion in this meta-analysis. The basic information about the included studies is shown in Supplementary Table 1. All the participants were diagnosed with MDD through a combination of DSM criteria and HAM-D scores. They were administered either oral zuranolone or a placebo once daily. A detailed quality assessment of the included literature is presented in Supplementary Table 1.

### 3.2 Primary outcomes

#### 3.2.1 HAM-D score on day 15

**FIGURE 2:**
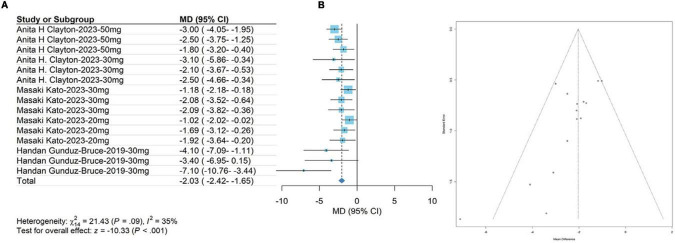
**(A)** denotes the HAM-D score on day 15; **(B)** denotes the funnel plot.

The meta-analysis results showed that the HAM-D scores in the treatment group were significantly higher than those in the control group at day 15 (WMD, −2.03; 95% CI, −2.42 to −1.65; *P* < 0.001). The heterogeneity was low (*χ2* = 21.43; *P* = 0.09; *I*^2^= 35.0%). The results are shown in Figure 2A. The funnel plot (in Figure 2B) shows a visual assessment of potential publication bias. Egger’s test showed that the results were not significantly affected by publication bias (*t* = −3.06; *P* = 0.0092).

#### 3.2.2 Response and remission rate on day 15 of HAM-D score

**FIGURE 3:**
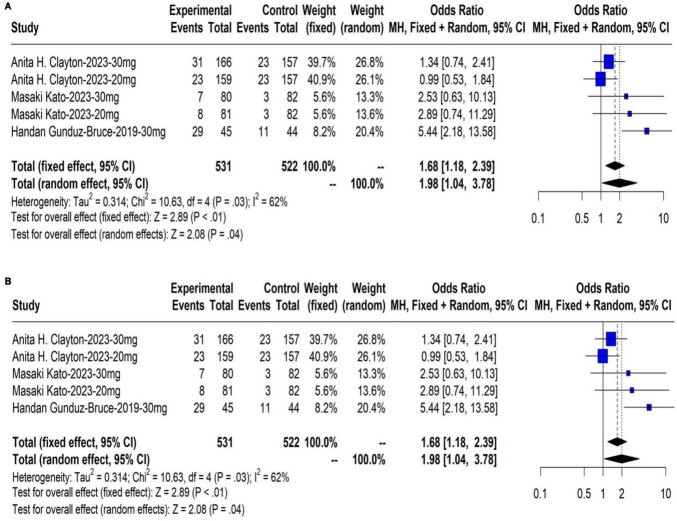
**(A)** The response rate on day 15 of HAM-D score; **(B)** The remission rate on day 15 of HAM-D score.

The meta-analysis results showed that the response rate of HAM-D score in the treatment group were significantly higher than those in the control group at day 15 (OR: 1.46, 95% CI: 1.11 to 1.92, *P* < 0.01). The results are shown in Figure 3A. The meta-analysis results showed that the remission rate of HAM-D score in the treatment group were significantly higher than those in the control group at day 15 (OR: 1.68, 95% CI: 1.18 to 2.39, *P* < 0.01). The results are shown in Figure 3B.

### 3.3 Secondary outcomes

#### 3.3.1 HAM-A score on day 15

**FIGURE 4:**
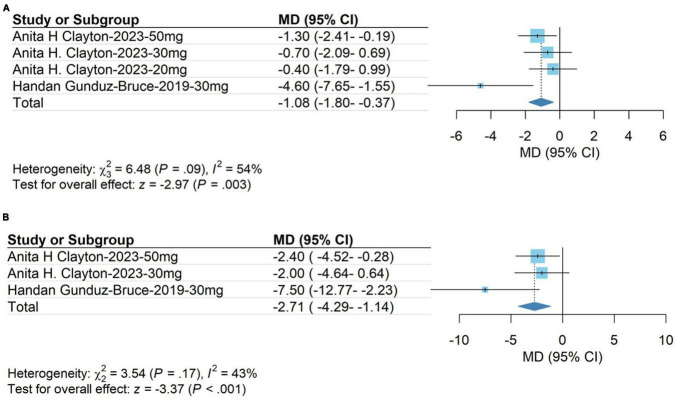
**(A)** HAM-A score on day 15; **(B)** Montgomery-Asberg Depression Rating Scale (MDARS) score on day 15.

The meta-analysis results showed that changes in HAM-A scores on day 15 in the group receiving zuranolone were significantly higher than those in the control group (WMD, −1.08; 95% CI, −1.80 to −0.37; *P* = 0.003). The heterogeneity was low (*χ2* = 6.48; *P* = 0.09; *I*^2^ = 54.0%). Egger’s test showed that the results were not significantly affected by publication bias (*t* = −1.58; *P* = 0.2546). The results are shown in Figure 4A.

#### 3.3.2 Montgomery-Asberg depression rating scale score on day 15

The meta-analysis results showed that the Montgomery-Asberg Depression Rating Scale (MDARS) score in the treatment group was significantly higher than that in the control group on day 15 (WMD, −2.71; 95% CI, −4.29 to −1.14; *P* < 0.001). The heterogeneity was low (*χ2* = 3.54; *P* = 0.17; *I*^2^ = 43.0%). Egger’s test showed that the results were not significantly affected by publication bias (*t* = −2.35; *P* = 0.2560). The results are shown in Figure 4B.

### 3.4 Subgroup analyses

Subgroup analyses of the primary outcomes were conducted, and the results showed that most subgroups yielded consistent results. There were no significant differences among the subcategories within each subgroup.

#### 3.4.1 HAM-D score of 30 mg zuranolone on days 3, 8, and 15

**FIGURE 5:**
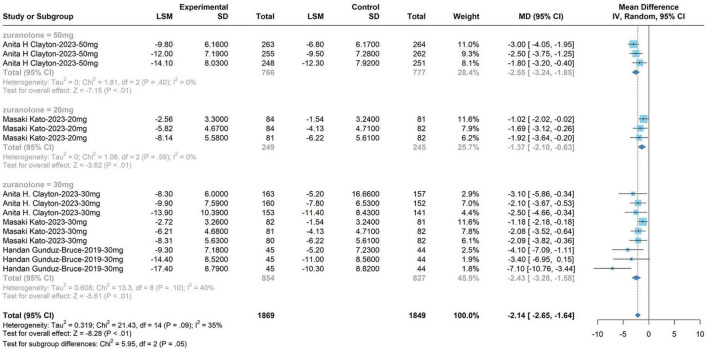
Hamilton Depression Rating Scale (HAM-D) score of 30 mg zuranolone on days 3, 8, and 15.

The subgroup analysis for the 30 mg zuranolone dosage yielded the following outcomes: WMD, −2.55; 95% CI, −3.24 to −1.58; *P* = 0.05; *I*^2^ = 35%; fixed model. The meta-analysis demonstrated alterations in zuranolone’s HAM-D score at day 3 (WMD, −2.55; 95% CI, −3.20 to −1.00; *P* < 0.01; *I*^2^ = 66%; fixed model), changes in zuranolone’s HAM-D score at day 8 (WMD, −2.17; 95% CI, −2.86 to −1.48; *P* < 0.01; *I*^2^ = 0%; fixed model), and modifications in zuranolone’s HAM-D score on day 15 (WMD, −2.47; 95% CI, −3.64 to −1.29; *P* < 0.01; *I*^2^ = 46%; fixed model). These results are shown in Figure 5.

#### 3.4.2 HAM-D score on day 15 for zuranolone doses of 20, 30, and 50°mg

**FIGURE 6:**
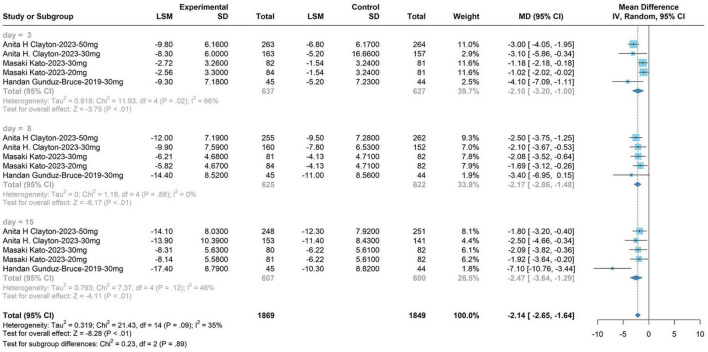
The dose of zuranolone was categorized into three groups: 50, 20, and 30 mg (HAM-D scores on day 15).

The dose of zuranolone was categorized into three groups: 50, 20, and 30 mg (HAM-D scores on day 15). Subsequently, the dose of zuranolone was analyzed within these subgroups (WMD, −2.55; 95% CI, −3.24 to −1.58; *P* = 0.05; *I*^2^= 35%; fixed model). The meta-analysis results showed changes in HAM-D scores in the 50 mg zuranolone group on day 15 (WMD, −3.49; 95% CI, −2.65 to −1.64; *P* = 0.05; *I*^2^ = 0%). Additionally, changes in HAM-D scores in the 20 mg zuranolone group on day 15 (WMD, −1.37; 95% CI, −2.10 to −0.63; *P* < 0.01; *I*^2^ = 0%, fixed model) and changes in HAM-D scores in the 30°mg zuranolone group on day 15 (WMD, −2.43; 95% CI, −3.28 to −1.58; *P* < 0.01; *I*^2^ = 40%; fixed model). The results are shown in Figure 6.

### 3.5 Adverse events during the treatment period

**FIGURE 7:**
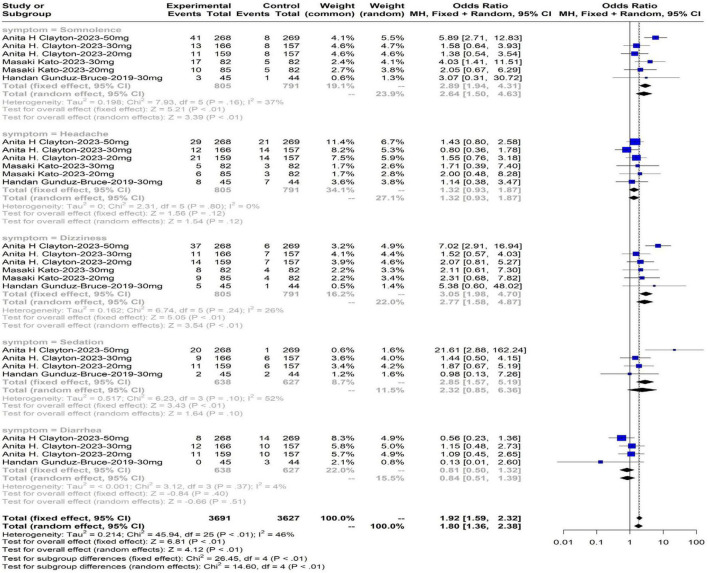
Adverse events.

The incidence rates of adverse reactions with zuranolone compared to placebo, ranked from highest to lowest, were as follows: dizziness at 3.05% (95% CI: 1.98 to 4.70), somnolence at 2.89% (95% CI: 1.94 to 4.31), sedation at 2.85% (95% CI: 1.57 to 5.19), headache at 1.32% (95% CI: 0.93 to 1.87), and diarrhea at 0.81% (95% CI: 0.50 to 1.32). Utilizing a fixed-effects model in a meta-analysis, a statistically significant discrepancy in adverse reaction occurrence rates emerged between the treatment and control groups (OR: 1.92, 95% CI: 1.59 to 2.32, *P* < 0.01). The results of the adverse reaction occurrence rates are shown in Figure 7, indicating an increased probability of adverse reactions following zuranolone administration.

### 3.6 Sensitivity analysis

Sensitivity analysis was performed on the HAM-D scores and the clinical efficacy of the zuranolone intervention for MDD. The sensitivity analyses indicated the robustness of all the findings. Consequently, one article was excluded, and a meta-analysis was conducted on the remaining articles. The combined results from the remaining studies remained statistically significant, underscoring the robustness of the findings and confirming that the exclusion had no impact on the final results. The results are presented in Supplementary Figure 1.

## 4 Discussion

Zuranolone afforded better efficacy than a placebo in HAM-D, HAM-A, and MDARS scores, and depressive response and remission rates. Notably, while HAM-A and MDARS scores exhibited improvement by day 15 compared to baseline, they displayed high heterogeneity due to missing scores on other treatment days in the clinical trial, precluding subgroup analysis. Consequently, our focus shifted to HAM-D scores on day 15 for subgroup analysis across zuranolone doses of 50, 20, 30, and 30 mg on various treatment days. Results indicated a delayed effect with the 20 mg dose, while no significant difference emerged between 30 and 50 mg, suggesting a plateau effect with increased dosage. Moreover, the 30 mg dose manifested rapid effectiveness on day 3, maintaining efficacy on days 8 and 15.

Additionally, a study (Clayton et al., 2023b) reported positive responses among patients in the zuranolone treatment group on day 15, maintaining an average of 86.1% improvement in HAM-D-17 on day 42 (4°weeks post-treatment). This underscores the need for further investigation into zuranolone’s long-term antidepressant effects and duration in future trials. Regarding adverse events, zuranolone showed an increased an incidence compared to placebo. Common adverse reactions encompassed dizziness, somnolence, sedation, headaches, and diarrhea. Literature (Kato et al., 2023) also reports occurrences of infection and invasion, rhinitis, neurological and gastrointestinal disorders, and skeletal diseases associated with zuranolone.

One of the earliest suggested biological mechanisms underlying MDD involves deficiencies in monoamine levels, such as 5-HT, noradrenaline, and dopamine (Hamon and Blier, 2013). The molecular mechanisms of MDD remain poorly understood. Studies have indicated that functional differences observed in fibroblasts derived from patients with MDD persist to some extent after reprogramming into induced NPCs, potentially linked to altered functioning of iPS neurons and thus possibly associated with the etiology of MDD (Fries et al., 2023). Furthermore, studies have revealed that MDD is associated with disruptions in various neurotransmitters within the brain, cerebrospinal fluid, and peripheral tissues (Pan et al., 2018), including imbalances in GABA (Fogaça and Duman, 2019).

Zuranolone is a neuroactive steroid (NAS) that acts as a positive allosteric modulator (PAM) of the GABAA receptor. GABA, a naturally occurring non-protein amino acid, serves as a vital inhibitory neurotransmitter in the mammalian CNS, with approximately 30% of the CNS synapses utilizing GABA as a transmitter. GABA plays an important role in various regions of the human brain, including the human cerebral cortex, hippocampus, thalamus, basal ganglia, and cerebellum, exerting regulatory influence on a variety of cognitive functions. Zuranolone is believed to function by restoring balance to brain networks responsible for critical functions like mood, arousal, behavior, and cognition. When GABA levels are deficient in the human body, it can lead to the manifestation of emotions such as anxiety, restlessness, fatigue, and worry. Zuranolone plays a role in helping to restore the proper functioning of dysfunctional GABAA receptors, potentially ameliorating these symptoms.

The study has several limitations. First, some of the included studies did not clearly report blinding and allocation concealment, which may have introduced heterogeneity. Second, in future research, it would be beneficial to include more studies in subgroup analysis to further validate the conclusions drawn in this study. Third, the limited number of original studies available for inclusion in this analysis increases the risk of false-positive results. Fourth, the published randomized controlled trials lacking the efficacy of zuranolone versus other antidepressants are still lacking in the literature, and subsequent research work should be aimed at addressing this. Given the limitations of these studies, multicenter, large-sample, double-blind, high-quality randomized controlled studies are needed to provide a higher level of evidence.

Zuranolone has received fast-track and breakthrough therapy designations from the FDA for treating MDD. Despite conducting three phase III clinical trials focusing on MDD, the MOUNTAIN study failed to meet the primary clinical endpoint of the Hamilton Depression Rating Scale assessment, showing only a 1.3 improvement compared to placebo, falling short of the generally considered clinically meaningful level of depression, set at 1.5. The WATERFALL study did achieve the primary clinical endpoint but exhibited only a 1.7 improvement compared to placebo. These trial outcomes, along with safety concerns raised by the FDA, included reports of suicidal ideation and behavior in clinical MDD studies. Regarding safety issues, Sage spokesman Matthew Henson mentioned that these concerns were associated with patients receiving the oral solution of Zurzuvae, not the approved capsule formulation. Henson emphasized the absence of reports regarding loss of consciousness among participants in studies for PPD and MDD. In reviewing the new FDA documents, RBC Capital Markets analyst Brian Abrahams highlighted an incident where a patient with MDD showed no response to stimuli for up to 50 min after receiving a high dose of Zurzuvae. Notably, this patient had taken a dose exceeding the currently approved dosage by 30 to 50%, experiencing loss of consciousness twice, while another subject experienced nearly 5°h of unconsciousness.

Despite these limitations, the quantitative meta-analysis of zuranolone in the treatment of MDD exhibited good result stability in sensitivity analysis. Furthermore, this study contributes to our understanding of the efficacy and safety of zuranolone intervention for MDD, highlighting improvements in the depressive state for patients with MDD.

Failure in clinical trials does not necessarily equate to failure in a drug’s potential indication. Clinical setbacks often stem from trial design or patient population. Adjustments in these areas can sometimes lead to the reintroduction of a clinically unsuccessful drug to the market. Numerous examples in various fields of drug development attest to this. In the case of Zuranolone for MDD, Sage remains in ongoing discussions with the FDA. Even if a drug is not ultimately deemed suitable for a particular indication, it does not signify complete failure, as there might be prospects for its use in other indications. Despite these limitations, the quantitative meta-analysis of zuranolone for severe depression demonstrates consistent and stable results in sensitivity analysis. This study significantly contributes to our understanding of the efficacy and safety of intervening with MDD, highlighting its efficacy in alleviating depressive symptoms among patients with MDD.

## Data availability statement

The original contributions presented in this study are included in this article/Supplementary Material, further inquiries can be directed to the corresponding author.

## Author contributions

WL designed the study. SW and WZ performed the search and analysis. ZL, YW, and TZ checked the analyzed the data. SW wrote the manuscript in consultation with WZ, ZL, YW, and TZ. All authors contributed to the article and approved the submitted version.

## Supplementary material

Supplementary Figure 1 Sensitivity analysis.

Supplementary Figure 2 Risk of bias summary.
