# Supplementary figures and images for: Efficacy and safety of zuranolone in the treatment of major depressive disorder: a meta-analysis
Source: Front Neurosci. 2024 Jan 16;17:1332329. doi: 10.3389/fnins.2023.1332329 (PMC10824890; doi:10.3389/fnins.2023.1332329)

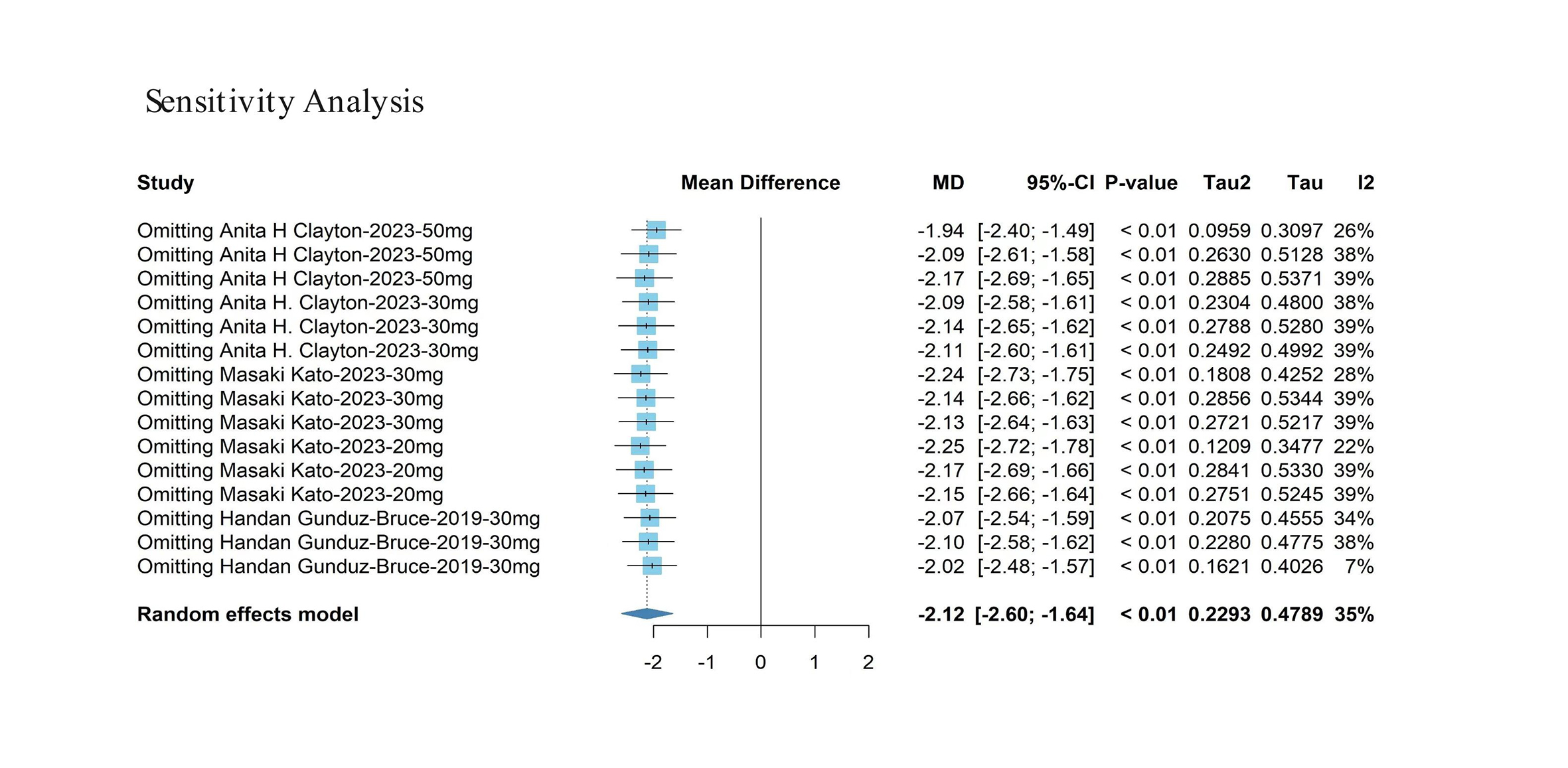

Supplement: Supplementary Figure 1 — Sensitivity analysis. [file Image_1.jpg]

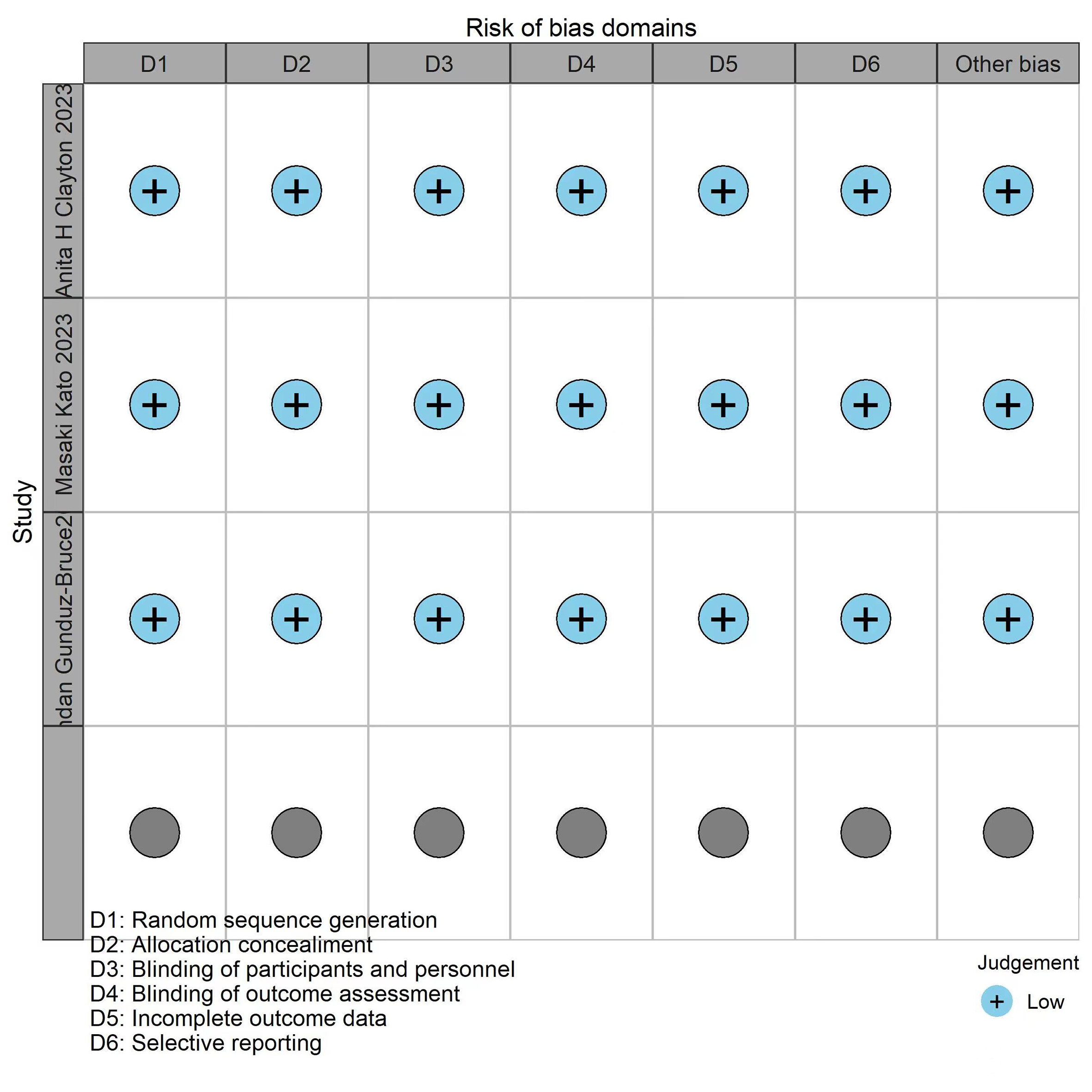

Supplement: Supplementary Figure 2 — Risk of bias summary. [file Image_2.jpg]
