# Supplementary material for: Efficacy and safety of zuranolone in the treatment of major depressive disorder: a meta-analysis
Source: Front Neurosci. 2024 Jan 16;17:1332329. doi: 10.3389/fnins.2023.1332329 (PMC10824890; doi:10.3389/fnins.2023.1332329)
Supplement: Supplementary file 5 [file Table_3.DOCX]

**Search strategies**

| **PubMed** | | |
| --- | --- | --- |
| Number | Query | Results |
| #1 | "Depressive Disorder, Major"[Mesh] | 38,976 |
| #2 | (((((((((((((Major Depressive Disorders[Title/Abstract]) OR (Major Depressive Disorder[Title/Abstract])) OR (Paraphrenia, Involutional[Title/Abstract])) OR (Involutional Paraphrenia[Title/Abstract])) OR (Involutional Paraphrenias[Title/Abstract])) OR (Paraphrenias, Involutional[Title/Abstract])) OR (Psychosis, Involutional[Title/Abstract])) OR (Involutional Psychoses[Title/Abstract])) OR (Involutional Psychosis[Title/Abstract])) OR (Psychoses, Involutional[Title/Abstract])) OR (Depression, Involutional[Title/Abstract])) OR (Involutional Depression[Title/Abstract])) OR (Melancholia, Involutional[Title/Abstract])) OR (Involutional Melancholia[Title/Abstract]) | 33,855 |
| #3 | #1 OR #2 | 52,578 |
| #4 | "zuranolone" [Supplementary Concept] | 25 |
| #5 | (((SAGE-217[Title/Abstract]) OR (CS-2797[Title/Abstract])) OR (BIIB125[Title/Abstract]) | 23 |
| #6 | #4 OR #5 | 38 |
| #7 | ((Randomized controlled trial[Publication Type]) OR (Randomized[Title/Abstract])) OR (Placebo[Title/Abstract]) | [1,030,670](https://pubmed.ncbi.nlm.nih.gov/?term=((Randomized+controlled+trial[Publication+Type])+OR+(Randomized[Title/Abstract]))+OR+(Placebo[Title/Abstract])&ac=no&sort=relevance) |
| #8 | #3 AND #6 AND #7 | 13 |
| **Embase** | | |
| Number | Query | Results |
| #1 | 'Depressive Disorder, Major'/exp | 48,320 |
| #2 | 'major depressive disorders':ab,ti OR 'major depressive disorder':ab,ti OR 'mdd' | 48,292 |
| #3 | #1 OR #2 | 75,914 |
| #4 | 'zuranolone'/exp | 145 |
| #5 | 'sage-217':ab,ti OR 'CS-2797':ab,ti OR 'BIIB125':ab,ti | 72 |
| #6 | #4 OR #5 | 170 |
| #7 | 'randomized controlled trial':ab,ti OR 'randomized':ab,ti OR 'placebo':ab,ti | 1,141,762 |
| #8 | #3 AND #6 AND #7 | 49 |
| **Cochrane** | | |
| Number | Query | Results |
| #1 | MeSH descriptor: [Depressive Disorder, Major] explode all trees | 6,571 |
| #2 | (Major Depressive Disorders):ti,ab,kw OR (Major Depressive Disorder):ti,ab,kw OR (Paraphrenia, Involutional):ti,ab,kw OR (Involutional Paraphrenia):ti,ab,kw OR (Involutional Paraphrenias):ti,ab,kw OR (Paraphrenias, Involutional):ti,ab,kw OR (Psychosis, Involutional):ti,ab,kw OR (Involutional Psychoses):ti,ab,kw OR (Involutional Psychosis):ti,ab,kw OR (Psychoses, Involutional):ti,ab,kw OR (Depression, Involutional):ti,ab,kw OR (Depression, Involutional):ti,ab,kw OR (Involutional Depression):ti,ab,kw OR (Melancholia, Involutional):ti,ab,kw OR (Involutional Melancholia):ti,ab,kw | 16,234 |
| #3 | #1 OR #2 | 16,234 |
| #4 | (zuranolone):ti,ab,kw | 31 |
| #5 | (sage-217):ti,ab,kw OR (CS-2797):ti,ab,kw OR (BIIB125):ti,ab,kw | 65 |
| #6 | #4 OR #5 | 85 |
| #7 | (randomized controlled trial):ti,ab,kw OR (randomized):ti,ab,kw OR (placebo):ti,ab,kw | 1,226,807 |
| #8 | #3 AND #6 AND #7 | 53 |
| **Web of Science** | | |
| Number | Query | Results |
| #1 | ((((((((((((((((TS=(Depressive Disorders, Major))) OR TS=(Major Depressive Disorders)) OR TS=(Major Depressive Disorder)) OR TS=(Paraphrenia, Involutional)) OR TS=(Involutional Paraphrenia)) OR TS=(Involutional Paraphrenias)) OR TS=(Paraphrenias, Involutional)) OR TS=(Psychosis, Involutional)) OR TS=(Involutional Psychoses)) OR TS=(Involutional Psychosis))) OR TS=(Psychoses, Involutional)) OR TS=(Depression, Involutional)) OR TS=(Involutional Depression)) OR TS=(Melancholia, Involutional)) OR TS=(Involutional Melancholia) | 38,345 |
| #2 | (((TS=(zuranolone)) OR TS=(sage-217)) OR TS=(CS-2797)) OR TS=(BIIB125) | 116 |
| #3 | ((TS=(randomized controlled trial)) OR TS=(randomized)) OR TS=(placebo) | 689,152 |
| #4 | #1 AND #2 AND #3 AND #4 | 26 |
